# *In vitro* digested ingredients as substitute for ileal digesta in assessing protein fermentation potential in growing pigs

**DOI:** 10.1017/S0007114525000108

**Published:** 2025-02-14

**Authors:** Hanlu Zhang, John W. Cone, Arie K. Kies, Wouter H. Hendriks, Nikkie van der Wielen

**Affiliations:** 1 Animal Nutrition Group, Department of Animal Sciences, Wageningen University & Research, Wageningen, The Netherlands; 2 State Key Laboratory of Animal Nutrition, College of Animal Science and Technology, China Agricultural University, Beijing, People’s Republic of China; 3 ArieKiesAdvies, Druten, The Netherlands; 4 Division of Human Nutrition and Health, Department of Agrotechnology and Food Sciences, Wageningen University & Research, Wageningen, The Netherlands

**Keywords:** Plant protein fermentation, Pig, *In vitro* digestion, Ileal digesta, Gas production

## Abstract

Understanding protein fermentation in the hindgut of pigs is essential due to its implications for health, and ileal digesta is commonly used to study this process *in vitro*. This study aimed to assess the feasibility of utilising *in vitro* digested residues as a replacement for ileal digesta in evaluating the protein fermentation potential. *In vitro* residues from cottonseed meal, maize germ meal, peanut meal, rapeseed cake, rapeseed meal, soyabean meal and sunflower meal were analysed using a modified gas production (GP) technique and curve fitting model to determine their fermentation dynamics and compare with the use of ileal digesta. Significant variations were observed in GP parameters between *in vitro* digested residues, indicating differences in nitrogen utilisation by fecal microbiota. Soyabean meal and sunflower meal exhibited the highest maximum GP rates (R_max_), with values of 29·5 ± 0·6 and 28·0 ± 1·2 ml/h, respectively, while maize germ meal showed slowest protein utilisation (17·3 ± 0·2 ml/h). A positive relationship was found between the R_max_ of *in vitro* residues and ileal digesta (R^2^ = 0·85, *P* < 0·01). However, GP potential (GP_s_) showed a tendency for a negative relationship (R^2^ = 0·39, *P* < 0·1), likely due to narrow observed GP_s_ values and the presence of varied endogenous proteins in ileal digesta. Our results demonstrate the potential of using *in vitro* digested residues as a substitute for ileal digesta in assessing the fermentation potential of protein ingredients, particularly regarding the rate of protein fermentation.

In the hindgut of animals, proteinaceous material either serves as building blocks for bacterial cells or enter catabolic pathways, supplying energy to gut bacteria in the absence of sufficient carbohydrates and producing various metabolites^(1)^. The destiny of endogenous and dietary undigested proteins, peptides and amino acids in the hindgut is influenced by factors such as diet, digestibility, endogenous secretions and microbial composition, illustrating the dynamic interplay between microbial processes and nutrient utilisation^(2)^. Furthermore, in the presence of sufficient fermentable carbohydrates, the availability of nitrogen (N) can influence the growth of the microbiota, consequently impacting the quantity of metabolites generated during fermentation.

Understanding gastrointestinal protein fermentation in nutrition is important given its potential unwanted effects on health^(3)^. *In vivo* research in humans and animals has predominantly focussed on single time point measurements of protein fermentation-associated metabolites, which is the net result of production, breakdown and absorption^(4)^. An *in vitro* approach can provide more detailed information on the degradation kinetics of substrate and synthesised metabolites compared to *in vivo* studies where the various processes are difficult to discern. Previous research utilising ileal digesta from pigs has provided valuable insights into the complexities of protein fermentation^(5,6)^. The requirement to obtain ileal digesta of animals in such studies still poses ethical and practical challenges^(7)^. The latter encourages the use of *in vitro* methodologies where there is no need for ileal cannulated pigs or sampling of digests under anaesthesia. An additional advantage of an *in vitro* approach is that it avoids the influence of endogenous proteinaceous components – such as enzymes and mucus – present in ileal digesta, allowing for a clearer assessment of dietary protein fermentation potential. In contrast, *in vivo* studies are complicated by the presence of endogenous proteins that contribute additional N to the ileal digesta, potentially altering fermentation dynamics by providing an alternative protein source for microbial activity. This makes it challenging to distinguish the specific effects of dietary proteins from those of endogenous components on gas production (GP) and other fermentation parameters. While *in vitro* studies allow for controlled kinetic analysis and specific insights into protein-associated factors, translating these findings to the *in vivo* context requires careful consideration due to inherent differences in microbial interactions and nutrient sources. However, endogenous proteins might still be present and could undergo digestion in *in vitro* methods due to added enzymes, although likely to a limited extent.

To establish a direct link between protein fermentation and dietary protein characteristics, the ‘undigested’ dry matter of *in vitro* digested feed ingredients has been used in some studies^(8,9)^ as previous research demonstrated a significant correlation between *in vivo* protein digestibility and an *in vitro* digestibility assay^(10,11)^. Building upon previous work,^(8)^ we developed a modified *in vitro* GP technique, using an N-free buffer with an excess of fermentable carbohydrates, making N limiting for microbial fermentation. This technique was combined with a novel curve fitting model to describe N availability in substrates for potential fermentation^(12)^. The model fitted to the cumulative GP generates parameters that serve as indicators to describe the kinetics of protein utilisation by the microbiota. Our previous study found that ileal digesta from pigs fed various dietary protein sources showed different *in vitro* GP dynamics, when the same amount of N was provided^(5)^. These data indicate that the hydrolysis of indigestible dietary and endogenous proteinaceous material derived from protein sources differ, which provides insights into their fermentation potential in the pig hindgut.

Here, we aimed to determine whether the fermentation potential of protein sources can be determined by using *in vitro* digested residues. The same protein sources as used in our previous *in vitro* fermentation study with ileal digesta were digested *in vitro*, and the undigested dry matter was used for *in vitro* protein fermentation.

## Materials and methods

### Protein sources

Samples were obtained from previously conducted studies investigating the digestibility of different batches, cultivars or processed dietary protein sources for porcine diets in Beijing, China^(13–18)^ and transported to Wageningen University (Wageningen, the Netherlands). The standardised ileal digestibility of protein (SID_pro_) was determined for each batch in those studies, and data were provided on the chemical composition of the protein sources.

In total, fifty-nine samples originated from seven separate studies including ten batches of cottonseed meal (CSM), eight batches of maize germ meal (MGM), seven batches of peanut meal, four batches of differently processed rapeseed cake (RSC), nine batches of rapeseed meal, twelve batches of soyabean meal (SBM) and nine batches of sunflower meal (SFM). Each ingredient was grown at a different location in China during various years, except for the SBM, of which five batches originated from the USA and Brazil. All samples were stored at −20°C until transport and upon arrival at Wageningen University at room temperature.

### In vitro digestion

Samples were *in vitro* digested with pepsin and pancreatin to simulate digestion in the stomach and small intestine using the method described previously^(10)^ with minor modifications (enzymes from different brand or Product No. were used). Briefly, an accurately weighed sample (∼10 g) was incubated in 250 ml 0·1M HCl with 5 g/l pepsin (P-7000 Sigma Chemical Co.). After 1·5 h, pH was neutralised with 50 ml of 0·5M NaHCO_3_, followed by 1·5 h incubation with 250 ml added 0·165M phosphate buffer, containing 2 g/l porcine pancreatin (P-1750, Sigma Chemical Co.) and 2 ml/L amylase (A-3176, Sigma Chemical Co.). All the incubation was under continuous stirring at 39°C. After incubation, the fluid was filtered through nylon gauze with pores of 40 μm using a vacuum pump. After sequential washing with 70 % ethanol and acetone, undigested residues on the filter were collected, freeze dried and residues weighed. The nitrogen level in the samples before and after digestion was determined with the Dumas method (ISO 16634). *In vitro* protein digestibility (IVD_pro_) was calculated according to the difference in N content.

### Particle size determination

Particle size distribution was not determined for all protein sources due to limited sample quantities. The particle size of ten batches of CSM, six batches of SFM, four batches of peanut meal and three batches of RSC was determined by dry sieving in duplicate. The dry sieving was conducted using a sieve tower of six sieves (2·5, 1·25, 0·63, 0·315, 0·16 and 0·071 mm) and a pan. An accurately weight amount of sample (∼100 g) was placed on the top sieve (2·5 mm) of the sieve tower which was located in a shaker (AS 200 Control, Retsch, Haan, Germany) employing a 3-D throwing motion for 10 min with an amplitude of 2 mm and an interval shaking time of 6 s. Each sieve and the pan was accurately weighed and the weight of the sample in each sieve calculated. The particle size distribution was determined by calculating the geometric mean diameter and geometric standard deviation according to Wilcox *et al.* ^(19)^.

### In vitro protein fermentation

For fermentation, a precisely weighed amount of *in vitro* residue containing 10 mg N was incubated in three independent runs. Blank bottles without substrate as well as bottles containing intact whey protein isolate (WPI, Fonterra) were included in each run as controls. The *in vitro* protein fermentation procedure was performed as described by Zhang et al^(12)^. Briefly, sealed bottles of 250 ml containing 60 ml of 2 % pig fecal inoculum (prepared from the same batch of frozen inoculum sourced from twenty growing pigs, as in our previous study^(12)^) in an N-free buffer were prepared at the start of each run and incubated at 39°C until the addition of the test substrate. The timing of the addition of substrate to the buffer–fecal mixture was determined by monitoring the GP of the blank bottles at 39°C, which contained the same buffer–fecal mixture. This blank GP was recorded continuously using the method described by Cone *et al.* ^(8,20)^, until it reached a plateau after 1–2 h. Subsequently, the *in vitro* residue and control substrates were added to the different bottles and incubated in water baths at 39°C for 48 h, with continuous recording of GP. The water level in the water baths was maintained throughout the fermentation period.

The buffer was supplemented with 21·56 g/l easily fermentable carbohydrates, namely 8·6 g maltose (M5885), 4·32 g pectin from citrus peel (P9135), 4·32 g xylose (X1500, all from Sigma-Aldrich) and 4·32 g soluble potato starch (Paselli WA4, Avebe food).

### Curve fitting

An updated Groot model^(21)^ described in our previous study^(12)^ was used to fit the GP curves. For each bottle, the following parameters were calculated or estimated: 1. lag time (T_lag_, h) of the start of fermentation (the time at which the cumulative GP of the substrate surpassed the cumulative GP of the blank within a run), 2. maximum GP rate (R_max_, ml/h) by dividing the gas released between two consecutive recorded time points by the time interval, 3. time when R_max_ occurred (T_Rmax_, h), 4. total GP generated from the protein source provided (GP_s_, ml/10 mg N) as determined by the model shown below, 5. time when GP_s_ occurred and microbiota turnover is assumed to start (T_GPs_, h) and 6. slope (ml/h) of the linear line fitted to the cumulative GP after T_GPs_.

Model used to fit the cumulative GP data of individual bottles:

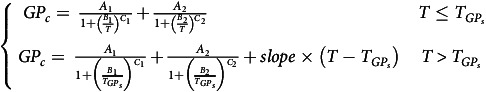

where GP_c_ (ml/10 mg N) denotes the amount of gas produced per 10 mg N of sample incubated (corrected by the GP of the blank groups at T_lag_) at time T after T_lag_, A_i_ (ml/10 mg N) represents the asymptotic GP, B_i_ (h) is the time after incubation at which half of the asymptotic amount of gas has been formed and C_i_ is a constant determining the sharpness of the switching characteristic of the curve. The parameter *i* indicates the number of phases in the curve (*i* = 1, 2). The model was used to derive GP_s_, T_GPs_ and the slope.

To further compare the GP potential of *in vitro* residues and their corresponding ileal digesta, all parameters were converted to the ratio relative to WPI in the same run. Data of ileal digesta were obtained from our previous study^(5)^.

### Statistical analyses

The values for T_lag_, R_max_, T_Rmax_, GP_s_, T_GPs_ and slope of *in vitro* digested ingredients and WPI were analysed using a mixed model. In this model, protein source was considered as a fixed factor, and replication run was treated as a random factor, except when there was a run effect. Differences between individual protein sources and the positive control (WPI) were assessed using Dunnett’s test. To compare differences between protein sources, Tukey’s test was employed. Different batches within the same protein source were considered as nested factors when examining source effects. If the nested factor was found to be non-significant, it was removed, and the ingredient was then included as a random factor. Additionally, differences within protein sources were evaluated across different incubation runs for each batch, with replication run treated as random factor if there was no run effect. The residuals of each model were checked for normality and homoscedasticity using QQ plot. The GP parameters of the same substrate (WPI) used in the current study and our previous study^(5)^ were compared by t-test. After proving, there were no differences between studies, and linear regression correlation analysis was conducted to compare the GP parameters of the *in vitro* residue here and ileal digesta samples from our previous study^(5)^. The differences between IVD_pro_ and SID_pro_ within protein source were compared by a paired *t* test. Additional regression model was derived to predict the *in vitro* digestibility from the SID_pro_. All statistical analyses were conducted using SAS 9.4 (SAS Inst. Inc.), residues of all the GP parameters were normally distributed and probability values < 0·05 were considered significant. Group values were reported as means ± standard error.

## Results

### In vitro gas production

**Figure 1.**
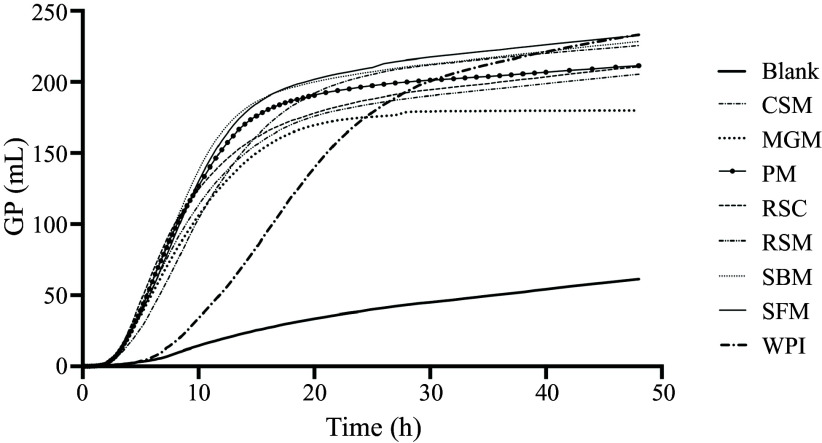
Mean of measured 48 h *in vitro* cumulative gas production (GP) of porcine fecal inoculum (Blank, *n* 3), seven *in vitro* digested protein sources and whey protein isolate (WPI, *n* 4). Protein sources include different batches of cottonseed meal (CSM, *n* 10), maize germ meal (MGM, *n* 8), peanut meal (PM, *n* 7), rapeseed cake (RSC, *n* 4), rapeseed meal (RSM, *n* 9), soyabean meal (SBM, *n* 12) and sunflower meal (SFM, *n* 9). All samples contained 10 mg of nitrogen and were incubated in 3 runs.

**Figure 2.**
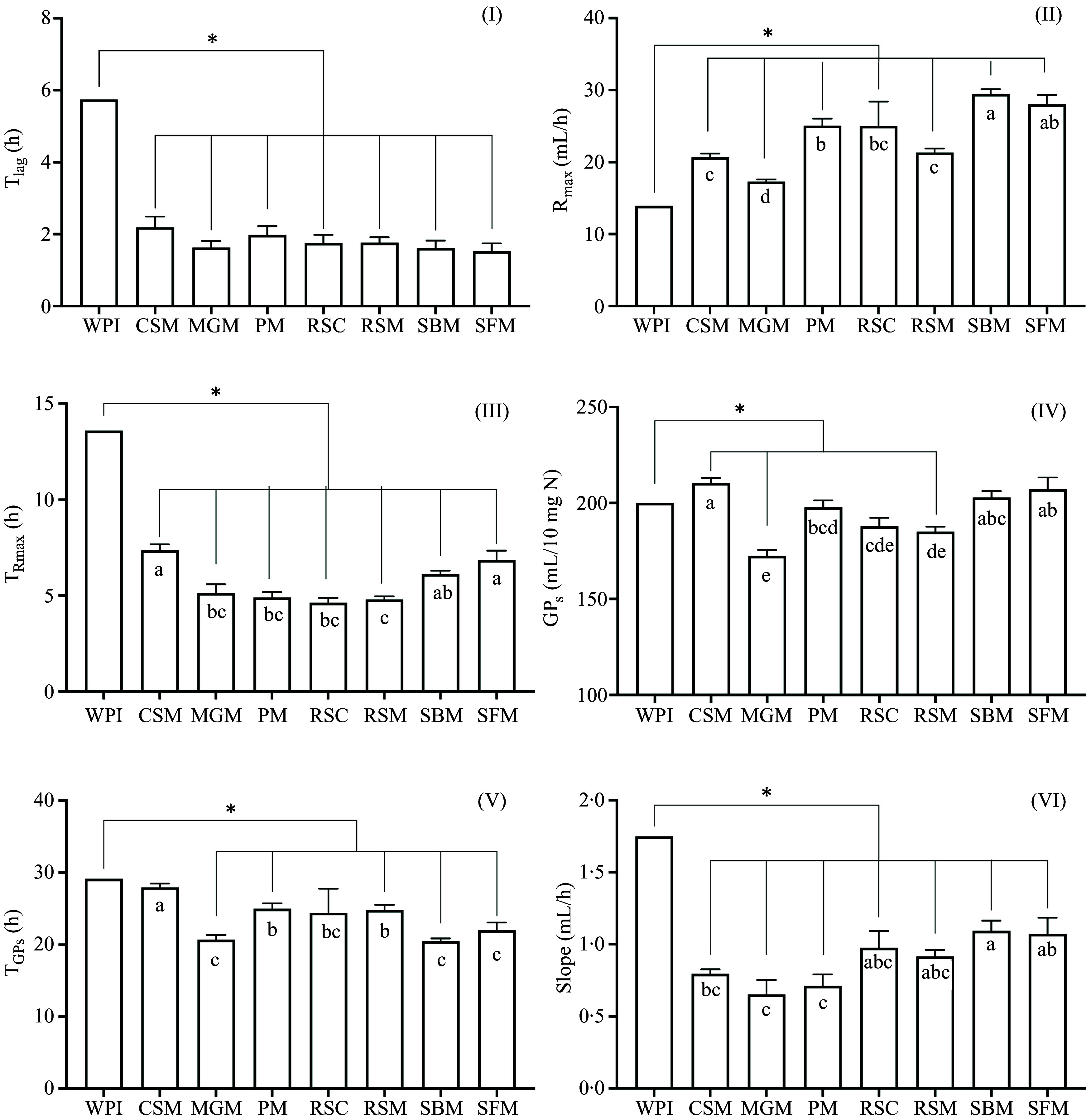
*In vitro* fermentation parameters of whey protein isolate (WPI) and seven *in vitro* digested protein sources containing 10 mg nitrogen. Lag time (T_lag_, I), maximum gas production rate (R_max_, II), time when maximum rate occurred (T_Rmax_, III), cumulative gas production of protein substrate determined by the model (GP_s_, IV), time when GP_s_ occurred (T_GPs_, V) and slope of the linear line of the model (slope, VI) during 3 incubation runs were shown (means (sem)). Protein sources include different batches from cottonseed meal (CSM, *n* 10), maize germ meal (MGM, *n* 8), peanut meal (PM, *n* 7), rapeseed cake (RSC, *n* 4), rapeseed meal (RSM, *n* 9), soybean meal (SBM, *n* 12) and sunflower meal (SFM, *n* 9). Within panel, bars with an asterisk differed (*P* < 0·05) to WPI, while bars with different letters indicate differences (*P* < 0·05) between *in vitro* digested protein source.

The measured cumulative GP curves of the blank of *in vitro* digested samples and of WPI over 48 h are shown in Figure 1. In general, the *in vitro* residues started to ferment (T_lag_) after 2 h, which was approximately 4 h earlier than WPI (Figure 2, *P* < 0·05). *In vitro* residues from six out of the seven protein sources also showed a higher R_max_ compared to WPI (13·9 ml/h, *P* < 0·05). The highest R_max_ was observed in the *in vitro* residue of SBM (29·5 ± 0·6 ml/h) and SFM (28·0 ± 1·2 ml/h). The *in vitro* residue of MGM (17·3 ± 0·2 ml/h) fermented significantly faster than WPI but significantly slower compared to the other protein sources. Moreover, T_Rmax_ for all the *in vitro* residues occurred earlier compared to WPI (average 5·7 ± 0·2 and 13·6 h, respectively, *P* < 0·05).

Gas production associated with the protein source (GP_s_) of all the groups except for MGM (173 ± 2·6 ml/10 mg N) and rapeseed meal (185 ± 2·6 ml/10 mg N) were close to WPI (200 ml/10 mg N) despite different R_max_ values. Among the *in vitro* digested samples, CSM produced the greatest amount of gas (210 ± 2·5 ml/10 mg N). Furthermore, *in vitro* residues of all the protein sources, except for CSM and RSC, reached GP_s_ earlier (T_GPs_, average 22·6 ± 1·0 h) compared to WPI (29·1 h, *P* < 0·05). Significant differences were found in the slope values, assumed to be due to microbiota turnover. Whey protein isolate showed a significantly higher value (1·75 ml/h) than all the *in vitro* residues, which ranged from 0·66 ± 0·09 ml/h for MGM to 1·1 ± 0·07 ml/h for SBM.

Fermentation parameters of *in vitro* residues of the individual batches within a protein source are shown in online Supplementary Figure 1–6. Significant differences were detected for T_lag_ within batches for SBM, R_max_ for MGM, peanut meal and RSC, GP_s_ for CSM and SFM, T_GPs_ for SFM and slope for RSC and SBM.

### Gas production of in vitro residues and corresponding ileal digesta

**Figure 3.**
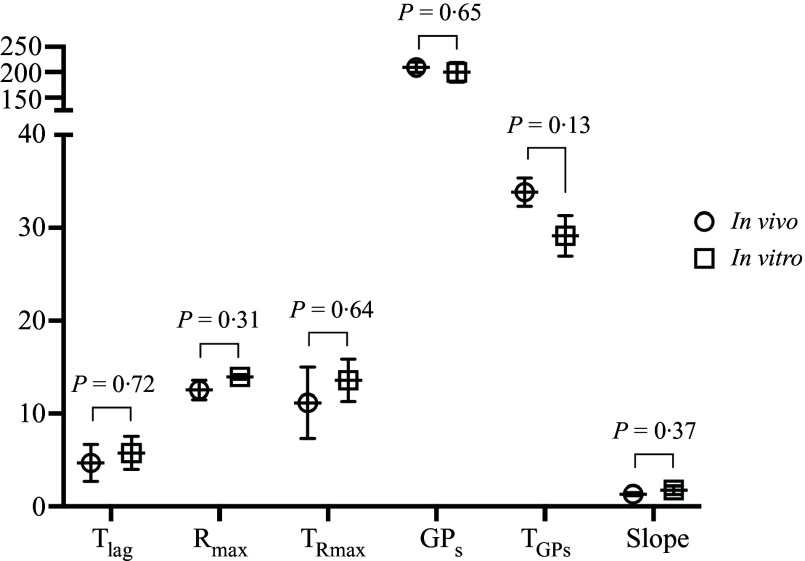
Comparison of gas production parameters for positive control group (whey protein isolate) in current study (*n* 3) and a previous study (*n* 4) in which ileal digesta from pigs, fed the same protein sources as used in current study, were fermented using an identical *in vitro* gas production technique^(5)^. Parameters include lag time (T_lag_, h), maximum gas production rate (R_max_, ml/h), time when maximum rate occurred (T_Rmax_, h), cumulative gas production of protein substrate determined by the model (GP_s_, ml/10 mg nitrogen), time when GP_s_ occurred (T_GPs_, h) and slope of the linear line of the model (slope, ml/h). Means (sem) during 3 or 4 incubation runs were shown, and *P* values were obtained by *t* test.

**Figure 4.**
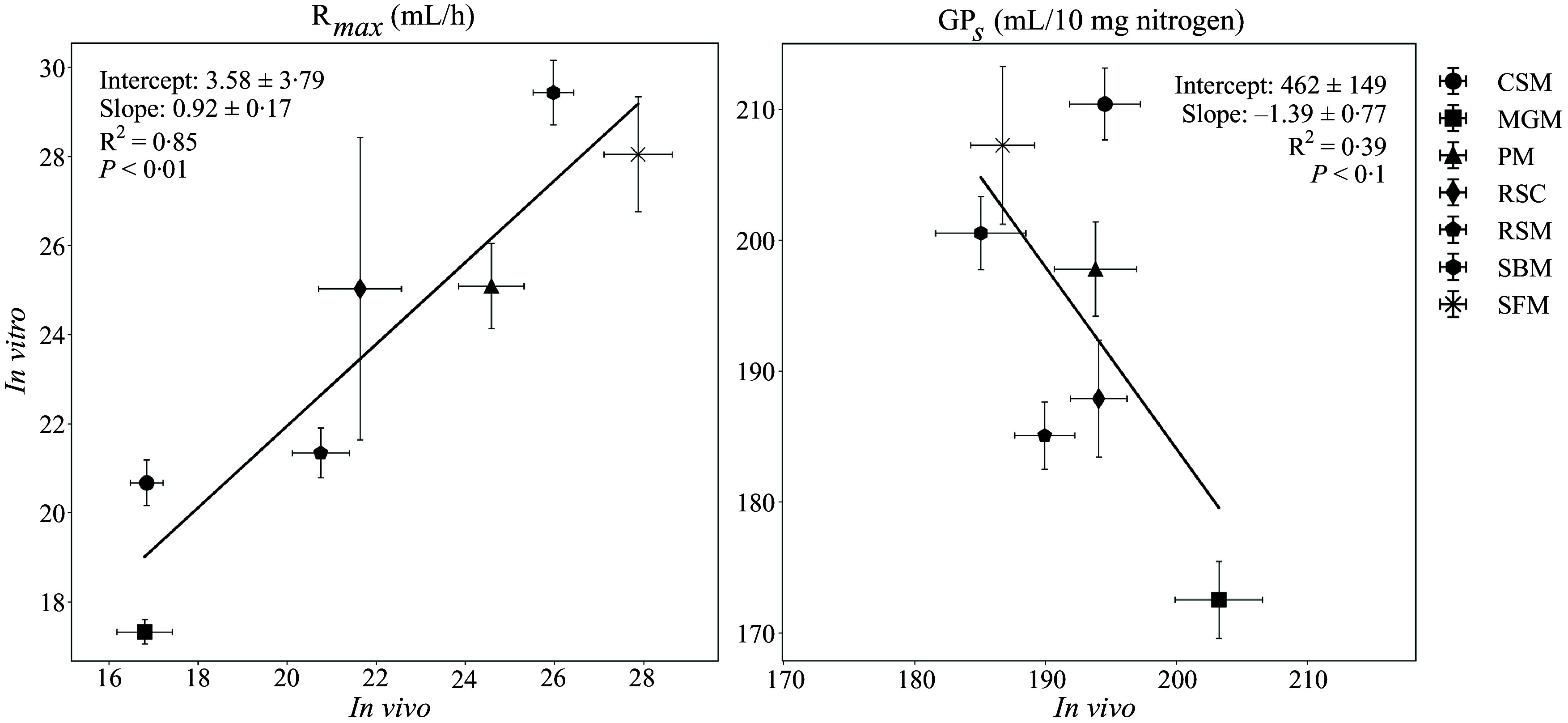
Linear regression of maximum gas production rate (R_max_) and cumulative gas production of protein substrate determined by the model (GP_s_) of porcine ileal digesta (*in vivo*) and their corresponding feed ingredients after *in vitro* digestion. Protein sources (with different batches) include cottonseed meal (CSM, *n* 10), maize germ meal (MGM, *n* 8), peanut meal (PM, *n* 7), rapeseed cake (RSC, *n* 4), rapeseed meal (RSM, *n* 9), soyabean meal (SBM, *n* 11) and sunflower meal (SFM, *n* 9). Mean (sem) in the plots) value of each protein source was used for the regression analysis.

For all the GP parameters, no significant differences were detected for the positive control (WPI) between the current study with undigested residues and our previous study with ileal digesta (Figure 3). Therefore, parameters of *in vitro* residues and corresponding ileal digesta were compared directly by linear regression analysis. A significant relationship (Figure 4) between the two types of substrates for R_max_ (R^2^ = 0·85, *n* 7, *P* < 0·01) was found while GP_s_ tended to have a negative relationship (R^2^ = 0·39, *n* 7, *P* < 0·1). No significant linear relationships were found for the other parameters.

### Crude protein content and digestibility

**Figure 5.**
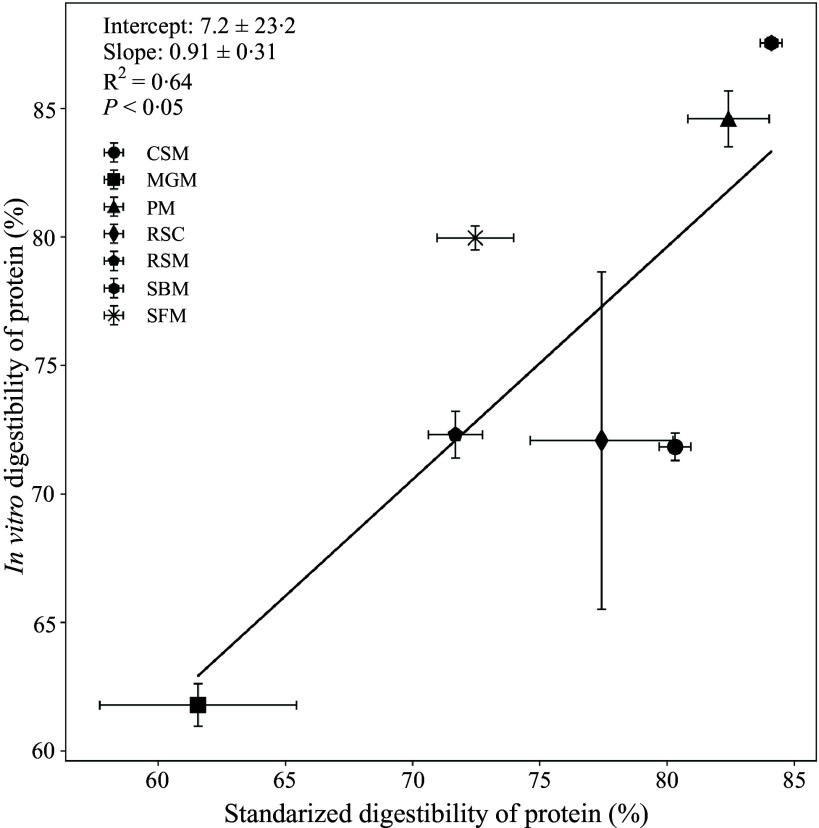
Linear relationship between *in vitro* digestibility and standardised ileal digestibility of protein across different sources. Protein sources (with different batches) include cottonseed meal (CSM, *n* 10), maize germ meal (MGM, *n* 8), rapeseed cake (RSC, *n* 4), peanut meal (PM, *n* 7), rapeseed meal (RSM, *n* 9), soyabean meal (SBM, *n* 12) and sunflower meal (SFM, *n* 9). Mean (sem) value of each protein source in the plot was used for the regression analysis.

The CP content of all the samples ranged from 21 % in MGM to 56 % in peanut meal (Table 1). Prior to fermentation, CP digestibility was assessed during *in vitro* digestion, revealing the lowest digestibility for MGM and the highest for SBM. A large variation in CP digestibility between batches was found for MGM (11 %, *in vivo*) and RSC (13 %, *in vitro*). Differences in the CP digestibility coefficients after *in vitro* digestion and *in vivo* digestion were observed for CSM, SBM and SFM (*P* < 0·01). The linear regression result between IVD_pro_ and the SID_pro_ of the 7 protein sources is shown in Figure 5. Overall, IVD_pro_ (%) = 7·2 + 0·91 × SID_pro_ (%), (R^2^ = 0·64, *n* 7, *P* < 0·05). Large variation within sources like RSC was observed.

**Table 1.** Standardised ileal (*in vivo*) and *in vitro* crude protein (CP) digestibility of various protein ingredients for growing pigs (Mean values and standard deviations)

Protein ingredient	Number of batches	CP^*^ (% dry matter)		CP digestibility (%)				Difference^‡^ (%)		*P* value
				*In vivo* ^†^		*In vitro*				
		Mean	sd	Mean	sd	Mean	sd	Mean	sd	
Cottonseed meal	10	52	5·8	80	2·0	72	1·7	8·5	2·7	< 0·01
Maize germ meal	8	21	1·0	62	11	62	2·3	–0·2	12	0·96
Peanut meal	7	56	2·7	82	4·2	85	2·9	–2·2	2·5	0·06
Rapeseed cake	4	41	2·8	77	5·6	72	13	5·4	17	0·58
Rapeseed meal	9	42	1·4	72	3·2	72	2·7	–0·6	2·5	0·47
Soyabean meal	12	51	2·2	84	1·7	88	0·8	–3·5	1·6	< 0·01
Sunflower meal	9	34	3·8	73	4·3	80	1·4	–7·5	5·0	< 0·01

* Crude protein content of ingredients before *in vitro* digestion, calculated by nitrogen level (measured by Dumas) multiplied with 6·25.

† Data from previous studies^(13–18)^.

‡ Between *in vivo* and *in vitro*.

Further linear regression analysis showed that R_max_ was positively related to *in vitro* crude protein digestibility: R_max_ = −9·75 + 0·44 × IVD_pro_, (R^2^ = 0·66, *P* < 0·01). Dry sieving results showed that the geometric mean diameter ± geometric standard deviation of tested samples ranged from 0·20 ± 0·13 for SFM to 0·32 ± 0·16 mm for CSM (online Supplementary Table S1). No significant correlation was found for IVD_pro_ and geometric mean diameter, both within and between protein sources.

## Discussion

To rank the fermentation potential of different protein sources, we used average values across batches for each source to capture an overall profile of each source’s fermentability. In the current study, distinct *in vitro* GP curves were observed for the *in vitro* digestion residues of different protein sources, indicating variability in the utilisation of N by the fecal microbiota. This variability is likely influenced by the type of N-containing molecules being present, their availability for absorption including hydrolysis and the subsequent utilisation of AAs in the metabolism. Significant variations were noted in the GP parameters between protein sources, notably R_max_ and T_Rmax_, indicating differences in the rate of hydrolysis among the residues following *in vitro* digestion. Protein sources such as SBM and SFM displayed both high R_max_ and GP_s_, indicating greater fermentation potential and suggesting they may more effectively support microbial activity in the gut. In contrast, MGM and CSM showed a lower R_max_, suggesting it ferments at a slower rate and may contribute less readily to microbial fermentation. This can be attributed to several factors, including a low protein solubility in MGM^(22)^, as well as the levels of anti-nutritional factors such as gossypol and the complex fibrous matrix present in CSM that limits their exposure to enzymes^(13,23)^. These factors likely contribute to a reduced rate of microbial protein hydrolysis, which in turn influences *in vitro* GP dynamics. This ranking provides valuable insights into how these protein sources could be selected and balanced within diets to optimise fermentation outcomes and gut health.

In addition, we aimed to compare our findings with previous data using ileal digesta of pigs fed identical protein sources^(5)^. Both our prior and present study highlight the sensitivity of the *in vitro* system to diverse protein sources, including variations between batches. Among these, R_max_ was shown as the most sensitive fermentation parameter, and the regression analysis underscores the potential of *in vitro* residues to predict ileal digesta. A significant positive relationship (R^2^ = 0·85, *n* 7, *P* < 0·01) was observed between R_max_ of the *in vitro* residues and those of the ileal digesta utilising the average values of each protein source. Notably, this relationship persisted despite the presence of endogenous protein in the latter but absent in the former. Therefore, it suggests that the proteinaceous material remaining after *in vitro* digestion and that present in ileal digesta exhibit similar hydrolysis rates by the fecal microbiota.

A tendency for a negative correlation was observed for GP_s_ between *in vivo* samples and *in vitro* digested samples (R^2^ = 0·39, *P* < 0·1). This is likely due to the narrow range (1·1 and 1·2-fold) of observed GP_s_ values for both sample types. This range is lower than other studies where a 1·4 or 1·8 fold difference was observed between different protein sources after *in vivo* or *in vitro* digestion^(6,8)^. The relative low variation observed here indicates that the microbiota were able to hydrolyse and subsequently metabolise the protein to the same extent but not at the same rate. Furthermore, the relation between *in vivo* undigested protein and *in vitro* digested protein sources in terms of GP_s_ is also affected by the presence of endogenous proteins, which can be present in varying amounts in the ileal digesta. As GP_s_ values for endogenous losses can differ^(12)^, variable amounts of endogenous N per 10 mg substrate N can affect GP_s_ values.

Although endogenous protein losses impact the comparison between *in vivo* samples (ileal digesta) and *in vitro* digested residues, overall, the *in vitro* residues appear to predict the rate of protein hydrolysis (R_max_) by the microbiota for ileal digesta samples. Interestingly, our study also revealed a relationship between R_max_ and IVD_pro_, with a slope of 0·44. This indicates that a higher digestible protein source is linked to a greater rate of microbial fermentation of the undigested residue. This relationship shows that proteinaceous material in *in vitro* residues is more readily hydrolysed by microbial enzymes when the digestibility is higher. While this may partly be due to smaller molecular size, it could also result from a more open or accessible protein matrix structure, allowing enzymes greater access to the substrate. This structural factor, along with molecular size, likely contributes to the enhanced hydrolysis efficiency observed in more digestible proteins, as demonstrated for WPI in a previous study^(12)^.

The IVD_pro_ of four out of the seven protein sources in the current study showed no differences with the corresponding SID_pro_ values obtained from growing pigs. After grouping the dataset by sources (*n* 7), a positive relationship with an R^2^ of 0·64 was found between IVD_pro_ and SID_pro_. This finding falls within the range reported by previous studies comparing *in vivo* and *in vitro* digestion^(10,24)^. The significant relationship suggests the possibility of using *in vitro* digestion to predict protein digestibility in animals at the source level. Nevertheless, within different sources, the values may not always align and can vary significantly between batches. The large variation found for IVD_pro_ of RSC is due to the relatively small sample size (*n* 4), meaning that a batch with a notably low value was not excluded as an outlier. One potential reason for SBM and SFM to differ (higher IVD_pro_) could be that during digestion, the reduced particle size can have a significant impact on digestibility,^(25)^ which can be due to the increased surface area to volume ratio, exposing more nutrients to digestive enzymes^(26)^. Therefore, it was recommended to report particle size distribution when using the *in vitro* digestibility assay developed by Boisen and Fernández^(25)^. However, no significant relationships between particle size (GMD) and IVD_pro_ were observed in the current study. Other underlying factors as mentioned previously, such as protein solubility and gossypol level, still may contribute to the lower IVD_pro_ compared to SID_pro_ for CSM. Collectively, these factors may pose challenges for the simplified *in vitro* environment (only two types of enzymes were added) to efficiently access the protein substrates. Together with findings from other studies^(10,24)^, it is suggested that while *in vitro* methods show a promise for predicting protein digestibility, they may not always align with *in vivo* results and can vary significantly between batches among different protein sources.

Using *in vitro* digested residues can provide valuable insights into the potential of the undigested protein in ileal digesta to ferment *in vitro*, despite possible protein digestibility differences as the same amount of N (10 mg) was used in the *in vitro* fermentation assay. It is worth noting that endogenous protein was not corrected for in ileal digesta samples to compare the GP parameters. For future studies, incorporating endogenous protein at a specific ratio, based on associated SID_pro_ values, into *in vitro* digested samples could lead to a more precise estimation.

### Conclusion

This study is the first to compare the *in vitro* protein fermentability of undigested residues derived from *in vitro* digested ingredients with their corresponding ileal digesta. The results show that, despite of some inconsistency between *in vitro* and *in vivo* protein digestibility, there is a potential to utilise the *in vitro* digestion method to replace animal experiments when estimating the fermentation potential of protein ingredients, particularly regarding the rate of protein fermentation. This approach provides a valuable framework for ranking ingredients based on their fermentability, which could be used in dietary formulations aimed at optimising gut health and nutrient utilisation. Future research should refine this method to account for batch variability and to further explore the implications of protein fermentability *in vivo*.

## Supporting information

Zhang et al. supplementary material Zhang et al. supplementary material

## Supplementary material

For supplementary material/s referred to in this article, please visit https://doi.org/10.1017/S0007114525000108
