# Supplementary material for: In vitro digested ingredients as substitute for ileal digesta in assessing protein fermentation potential in growing pigs
Source: Br J Nutr. 2025 Jan 27;133(3):400–7. doi: 10.1017/S0007114525000108 (PMC11946045; doi:10.1017/S0007114525000108)
Supplement: Zhang et al. supplementary material [file S0007114525000108sup001.docx]

***In Vitro* Digested Ingredients as Substitute for Ileal Digesta in Assessing Protein Fermentation Potential in Growing Pigs**

Hanlu Zhang^a,b^, John W. Cone^a^, Arie K. Kies^c^, Wouter H. Hendriks^a^ and Nikkie van der Wielen^a,d,*^

^a^ Animal Nutrition Group, Department of Animal Sciences, Wageningen University & Research, Wageningen, The Netherlands;

^b^ State Key Laboratory of Animal Nutrition, College of Animal Science and Technology, China Agricultural University, Beijing, China;

^c^ ArieKiesAdvies, Druten, The Netherlands;

^d^ Division of Human Nutrition and Health, Department of Agrotechnology and Food Sciences, Wageningen University & Research, Wageningen, The Netherlands.

**^*^**Correspondence: [nikkie.vanderwielen@wur.nl](mailto:nikkie.vanderwielen@wur.nl)

**Running title:** *In Vitro* Digestion for Pig Protein Fermentation

**Keywords:** Plant protein fermentation, pig, *in vitro* digestion, ileal digesta, gas production

| Supplemental Table 1. Particle size distribution (%; mean ± SD) and geometric mean diameter (GMD) and geometric standard deviation (GSD) for batches of cottonseed meal, peanut meal, rapeseed cake and sunflower meal. | | | | |
| --- | --- | --- | --- | --- |
| Sieve opening (mm) | Cottonseed meal  (n=10) | Peanut meal  (n=4) | Rapeseed cake  (n=3) | Sunflower meal  (n=6) |
| 2.5 | 0 | 0 | 0 | 0.2 ± 0.3 |
| 1.25 | 1.6 ± 0.3 | 0.6 ± 0.1 | 1.1 ± 0.2 | 1.1 ± 0.3 |
| 0.63 | 18.6 ± 5.7 | 4.4 ± 1.2 | 2.5 ± 1.5 | 3.1 ± 4.7 |
| 0.315 | 25.0 ± 4.9 | 30.5 ± 3.5 | 24.2 ± 11.4 | 15.5 ± 7.5 |
| 0.16 | 24.2 ± 2.2 | 23.5 ± 1.3 | 45.9 ± 9.1 | 27.5 ± 4.7 |
| 0.071 | 19.0 ± 4.4 | 19.9 ± 1.2 | 27.7 ± 7.6 | 35.2 ± 16.5 |
| Pan | 13.5 ± 6.0 | 21.5 ± 4.6 | 0.5 ± 0.2 | 18.9 ± 14.4 |
| GMD ± GSD | 0.32 ± 0.16 | 0.27 ± 0.15 | 0.23 ± 0.14 | 0.20 ± 0.13 |

Supplemental Figure 1. Lag time (T_lag_) during the *in vitro* incubation of *in vitro* digested protein sources (n=3) containing 10 mg nitrogen. Protein sources include cottonseed meal (CSM), maize germ meal (MGM), peanut meal (PM), rapeseed cake (RSC), rapeseed meal (RSM), soybean meal (SBM) and sunflower meal (SFM). Bars with different letters showed significant differences between least square means of different batches of one protein source (*P* < 0.05). Values are presented as means ± SEM.

Supplemental Figure 2. Maximum gas production rate (R_max_) during the *in vitro* incubation of *in vitro* digested protein sources (n=3) containing 10 mg nitrogen. Protein sources include cottonseed meal (CSM), maize germ meal (MGM), peanut meal (PM), rapeseed cake (RSC), rapeseed meal (RSM), soybean meal (SBM) and sunflower meal (SFM). Bars with different letters showed significant differences between least square means of different batches of one protein source (*P* < 0.05). Values are presented as means ± SEM.

Supplemental Figure 3. Time when maximum rate occurred (T_Rmax_) during the *in vitro* incubation of *in vitro* digested protein sources (n=3) containing 10 mg nitrogen. Protein sources include cottonseed meal (CSM), maize germ meal (MGM), peanut meal (PM), rapeseed cake (RSC), rapeseed meal (RSM), soybean meal (SBM) and sunflower meal (SFM). Values are presented as means ± SEM.

Supplemental Figure 4. Cumulative gas production of protein substrate determined by the model (GP_s_) during the *in vitro* incubation of *in vitro* digested protein sources (n=3) containing 10 mg nitrogen (N). Protein sources include cottonseed meal (CSM), maize germ meal (MGM), peanut meal (PM), rapeseed cake (RSC), rapeseed meal (RSM), soybean meal (SBM) and sunflower meal (SFM). Bars with different letters showed significant differences between least square means of different batches of one protein source (*P* < 0.05). Values are presented as means ± SEM.

Supplemental Figure 5. Time when cumulative gas production of protein substrate determined by the model occurred (T_GPs_) during the *in vitro* incubation of *in vitro* digested protein sources (n=3) containing 10 mg nitrogen (N). Protein sources include cottonseed meal (CSM), maize germ meal (MGM), peanut meal (PM), rapeseed cake (RSC), rapeseed meal (RSM), soybean meal (SBM) and sunflower meal (SFM). Bars with different letters showed significant differences between least square means of different batches of one protein source (*P* < 0.05). Values are presented as means ± SEM.

Supplemental Figure 6. Slope of the linear line of the model during the *in vitro* incubation of *in vitro* digested protein sources (n=3) containing 10 mg nitrogen (N). Protein sources include cottonseed meal (CSM), maize germ meal (MGM), peanut meal (PM), rapeseed cake (RSC), rapeseed meal (RSM), soybean meal (SBM) and sunflower meal (SFM). Bars with different letters showed significant differences between least square means of different batches of one protein source (*P* < 0.05). Values are presented as means ± SEM.
